# (Mis-)use of standard Autopilot and Full Self-Driving (FSD) Beta: Results from interviews with users of Tesla's FSD Beta

**DOI:** 10.3389/fpsyg.2023.1101520

**Published:** 2023-02-23

**Authors:** Sina Nordhoff, John D. Lee, Simeon C. Calvert, Siri Berge, Marjan Hagenzieker, Riender Happee

**Affiliations:** ^1^Department Transport and Planning, Delft University of Technology, Delft, Netherlands; ^2^Department of Industrial and Systems Engineering, University of Wisconsin-Madison, Madison, WI, United States; ^3^Department Cognitive Robotics, Delft University of Technology, Delft, Netherlands

**Keywords:** Full Self-Driving (FSD) Beta, automated driving, traffic safety, human factors, mind-off driving

## Abstract

Tesla's Full Self-Driving Beta (FSD) program introduces technology that extends the operational design domain of standard Autopilot from highways to urban roads. This research conducted 103 in-depth semi-structured interviews with users of Tesla's FSD Beta and standard Autopilot to evaluate the impact on user behavior and perception. It was found that drivers became complacent over time with Autopilot engaged, failing to monitor the system, and engaging in safety-critical behaviors, such as hands-free driving, enabled by weights placed on the steering wheel, mind wandering, or sleeping behind the wheel. Drivers' movement of eyes, hands, and feet became more relaxed with experience with Autopilot engaged. FSD Beta required constant supervision as unfinished technology, which increased driver stress and mental and physical workload as drivers had to be constantly prepared for unsafe system behavior (doing the wrong thing at the worst time). The hands-on wheel check was not considered as being necessarily effective in driver monitoring and guaranteeing safe use. Drivers adapt to automation over time, engaging in potentially dangerous behaviors. Some behavior seems to be a knowing violation of intended use (e.g., weighting the steering wheel), and other behavior reflects a misunderstanding or lack of experience (e.g., using Autopilot on roads not designed for). As unfinished Beta technology, FSD Beta can introduce new forms of stress and can be inherently unsafe. We recommend future research to investigate to what extent these behavioral changes affect accident risk and can be alleviated through driver state monitoring and assistance.

## 1. Introduction

Tesla's Autopilot is among the most capable and discussed partially automated driving systems currently available to drivers in production vehicles throughout the world. In October 2020, Tesla launched its Full Self-Driving (FSD) Beta program, which is an SAE level 2 partially automated driving system extending the operational design domain (ODD) of standard Autopilot—highway—enabling the car to drive in the automated mode on non-highway roads under the constant supervision of human drivers. Currently, ~160,000 pre-selected owners and drivers have been given access to FSD Beta (Korosec, 2021; Lambert, 2022a,b; Quick, 2022). FSD Beta has been controversially described as an “experiment on public roads” (Petrova, 2022) given unsafe vehicle behavior (Lambert, 2020), which prompted the California Department for Motor Vehicles to re-assess the FSD Beta trial (Lambert, 2022a).

The introduction of partially automated cars can be associated with direct changes in driver state and behavior. The direct changes represent the short-term, intended, and engineering effects as a direct consequence of system use. These can relate to driver state changes (i.e., attention, situational awareness, workload, stress, and drowsiness) and decisions about action/performance changes (i.e., driving, system handling, and error) (Kulmala, 2010; Martens and Jenssen, 2012). Human factors researchers have long pointed to the “ironies of automation” (Bainbridge, 1983), changing the role of the human driver from manual to supervisory control and creating so-called “out-of-the-loop” issues (Saffarian et al., 2012). Theoretical assumptions and empirical evidence about the effect of partially automated driving on drivers' situational awareness and workload are mixed, with some studies expecting a decrease in situational awareness (Boelhouwer et al., 2019; White et al., 2019), and others reporting an increase in situational awareness (Endsley, 2017). A meta-analysis by De Winter et al. (2014) showed that adaptive cruise control (ACC) and highly automated driving (HAD; corresponding with conditionally automated driving) could both increase and decrease driver's situational awareness. The authors further revealed that HAD resulted in a substantial decrease in workload, while ACC reduced drivers' workload by only a small amount. Metz et al. (2021) have revealed an increase in driver fatigue during the drive with automated driving functions. Hardman (2021) revealed that Autopilot users reported feeling less mentally and physically strained during and after traveling with Autopilot. Where many studies report a reduced workload with automation, Stapel et al. (2019) found an increased objective workload, particularly in complex traffic with Tesla Autopilot.

The introduction of partially automated cars can also be associated with indirect changes in driver state and behavior. The indirect changes pertain to the unintended longer-term positive and negative behavioral changes of human drivers adapting to the system (Sagberg et al., 1997; Lee, 2008; Martens and Jenssen, 2012). They can offset or negate some of the intended benefits (Martens and Jenssen, 2012; Robertson et al., 2017). Unintended effects can pertain to changes in the driver's state, performance, and attitudes, such as trust, overreliance, acceptance, and rejection (Martens and Jenssen, 2012). Mis-calibrated trust can take the form of overtrust (trust is higher than what is required by the system) and promote misuse (i.e., overreliance on automation), whereas under-trust can promote disuse of automation (i.e., underutilization of automation) (Parasuraman and Riley, 1997; Lee, 2008). A common example of misuse is complacency, which occurs when drivers fail to monitor the system (Lee, 2008; Banks et al., 2018; Cotter et al., 2021). Other unintended reported behavioral effects were mode confusion (Endsley, 2017; Banks et al., 2018; Wilson et al., 2020), testing the limits of the ODD (Banks et al., 2018), using Autopilot in ODDs for which it was not intended (Kim et al., 2021), driver distraction, drowsiness, and the difficulty of handling unanticipated automation failures (Endsley, 2017), an inadequate mental model of the capabilities of the automation, and skill degradation (i.e., loss of manual control skills due to automation) (Saffarian et al., 2012). Other studies provided evidence for little differences between the self-reported secondary task engagement during manual and partially automated driving (Shutko et al., 2018; Nordhoff et al., 2021). Evidence as to whether secondary task engagement deteriorates drivers' take over performance is inconclusive (Lin et al., 2019).

Partially automated cars can also change the amount of traveling (exposure) and route choice (see Kulmala, 2010). Scientific evidence on the expected changes in the amount of traveling — increase, decrease, or no change in the vehicle miles traveled—associated with the use of automated cars is ambiguous. Automated vehicles are expected to increase the productive use of travel time, which might encourage people to accept longer commutes (Singleton, 2019). Studies have revealed an expected increase in the vehicle miles traveled (Schoettle and Sivak, 2015; Harb et al., 2018; Hardman et al., 2019; Gurumurthy and Kockelman, 2020; Perrine et al., 2020; Lehtonen et al., 2022). Other studies have found no increase in the vehicle miles traveled (Zmud et al., 2016), and others provided evidence for both an increase and decrease in the vehicle miles traveled due to using automated vehicles (Childress et al., 2015; Wadud et al., 2016).

### 1.1. Research gaps

It is assumed that automated cars will improve traffic safety, eliminating human error (Ye and Yamamoto, 2019; Tafidis et al., 2022). These assumptions tend to remain speculative and untested, ignoring the emergence of new types of unintended safety risks, which may offset the expected safety benefits (Casner and Hutchins, 2019; McWilliams and Ward, 2021; Malin et al., 2022; Tafidis et al., 2022). Except for social media content (e.g., anecdotes and YouTube videos) (Reddit, 2022a), little is known about the unintended and intended changes of driver state and (travel) behavior with FSD Beta engaged and how these compare to standard Autopilot.

Previous studies contributed to our understanding of driver behavior of partially automated driving in highway environments. FSD Beta extends the ODD from the highway to non-highway roads, including additional functionality such as automated lane changes, route navigation, and navigating highways. The knowledge obtained in the present study will provide valuable information for manufacturers and policymakers about driver state and behavior and new types of safety risks that may emerge with the introduction of automated driving on public roads in complex urban environments. By comparing driver state and behavior and the use of a new (FSD Beta) to a more mature system (Autopilot), the study generates in-depth knowledge about driver's general experiences, benefits and risks, intended, short-term, and unintended, long-term behavioral adaptations associated with using automated driving in both highway and city environments.

### 1.2. Research questions

The four main research questions that the present study aimed to address are as follows:

- Research question 1: What are driver's direct short-term behavioral adaptations of using Autopilot and FSD Beta?
- Research question 2: What are driver's indirect long-term behavioral adaptations of using Autopilot and FSD Beta?
- Research question 3: How do drivers place their eyes, hands, and feet when using Autopilot and FSD Beta?
- Research question 4: How does using Autopilot and FSD Beta affect driver's amount of traveling and route choice?

## 2. Method

### 2.1. Recruitment

Semi-structured interviews were conducted with participants of Tesla's FSD Beta program and users of standard Autopilot. The study was approved by the Human Research Ethics Committee of the Delft University of Technology in the Netherlands.

Participants of the FSD Beta program were selected by Tesla, providing early access to owners (e.g., presidents of Tesla Owner clubs) and to drivers with a high safety score (Korosec, 2021; Lambert, 2022a,b). They received the following instructions from Tesla via email prior to the use of FSD Beta:

*Full Self-Driving is in limited early access Beta and must be used with additional caution. It may do the wrong thing at the worst time, so you must always keep your hands on the wheel and pay extra attention to the road. Do not become complacent. When Full Self-Driving Beta is enabled, your vehicle will make lane changes off highway, select forks to follow your navigation route, navigate around other vehicles and objects, and make left and right turns. Use Full Self-Driving Beta only if you will pay constant attention to the road, and be prepared to act immediately, especially around blind corners, crossing intersections, and in narrow driving situations. Every driver is responsible for remaining alert and active when using Autopilot and must be prepared to take action at any time*.

*As part of receiving FSD Beta, your vehicle will collect and share VIN-associated vehicle driving data with Tesla to confirm your continued eligibility for FSD Beta feature. If you wish to be removed from the limited early access FSD Beta please email xxx*.

For this study, respondents using FSD Beta were targeted via specialized online communities and forums (i.e., Discord, Facebook, Twitter, Reddit, YouTube, Instagram, Tesla Motors Club, and Tesla Motors Forum). FSD Beta was only available to drivers of North America and Canada by the time the study was conducted. For this reason, the focus of the recruiting targeted but was not limited to these geographic locations. The ownership of a Tesla was subjectively evaluated using self-reported data pertaining to the model and frequency of use of the Tesla.

### 2.2. Procedure

Respondents were interviewed online using Zoom, recording both sound and vision. The interviews were based on a predefined protocol that consisted of open-ended and closed-ended questions. In order to reduce the subjectivity of interview research, an interview protocol was created on Qualtrics (www.Qualtrics.com), and a link to the questions was sent via the chat function of Zoom at the beginning of the interview. In this way, respondents could directly see the questions in front of them, being able to move independently to the next question. The main role of the researcher was to listen as respondents completed the questionnaire to reduce the risk of influencing respondents during the interview. In line with semi-structured qualitative interview research (Longhurst, 2003), however, the interviewer asked follow-up questions in order to get clarification on certain aspects raised by the respondents or explore new phenomena brought up by respondents that were initially not covered by the interview protocol. The interviewer also encouraged respondents to skip questions in case questions were already answered. As the questions were standardized and logically followed each other, the intervention of the researcher was limited.

The interview protocol was divided into two main parts. At the start of the interview, respondents were asked to provide their informed consent to participate in the study. The first part consisted of mostly open-ended questions, while the second part consisted mostly of closed-ended questions pertaining to respondents' socio-demographic profile and travel behavior (e.g., age, gender, the highest level of education, and frequency of the use of Autopilot and FSD Beta), and general attitudes toward traffic safety.

Subject to the analysis of the present paper were the questions Q1, Q4, Q24–Q26, and Q30–Q35, as shown in Table 1. The responses obtained for Q4 answered research questions 1, 2, and 4. The responses obtained for Q24–Q26 and Q30–Q35 provided answers to research question 3. Respondents were asked to answer every question specifically for Autopilot and FSD Beta, respectively, in order to reflect on the differences between the systems per question if differences existed.

**Table 1:** Questions subjected to the analysis.

**Question number**	**Question**
Q1	Do you have the Full Self-Driving Beta (FSD Beta) feature? (1 = Yes, 2 = No)
Q4	Please describe your experience with using Autopilot and FSD Beta and the benefits and risks associated with using it. Please explain your answer
**With the next section, we would like to explore how you typically use Autopilot and FSD Beta**	
Q24	How do you typically place your hands on the steering wheel when Autopilot and FSD Beta is active? Please select the image that serves as best representation of your placement of your hands on the steering wheel when Autopilot/FSD Beta is active and explain your answer Figure is from Morando et al. (2021)
	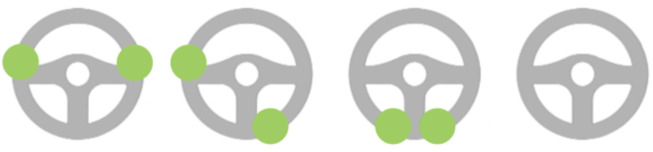
Q25	Do you typically keep your hands on the steering wheel at all times?
Q26	Are you typically fully attentive and alert at all times?
Q30	How do you typically place your eyes when Autopilot and FSD Beta is active?
Q31	Do you typically keep your eyes on the road at all times?
Q32	Do you typically monitor the vehicle and its surroundings at all times?
Q33	How do you typically place your feet when Autopilot and FSD Beta is active?
Q34	Do you typically stay prepared to take corrective actions at all times?
Q35	Has your use of Autopilot (in terms of how you placed your hands on the steering wheel, eyes on the road, and feet) changed over time? If so, how?

Table A1 in the Appendix presents an overview of the questions asked in the first part of the interview. Further questions will be processed in the following studies as it is beyond the scope of the present study to analyze the responses obtained for these questions in sufficient detail.

### 2.3. Data analysis

Following the procedure adopted in previous interview studies (Nordhoff et al., 2019; Doubek et al., 2021), the data analysis was performed in four steps by the first author of the present study:

1. First, the interviews were recorded using Zoom and transcribed verbatim using the transcription software of Microsoft Teams. The transcripts were compared with the audio files, and minor corrections were performed.
2. In the second step, Atlas.ti Version 22.0.2 was used to create main categories and sub-categories of and analyze the data. The main categories were developed deductively using the safety assessment framework by Kulmala (2010), which assumes that the introduction of Connected Intelligent Transport Systems (C-ITS) and new in-car technology is associated with direct modifications of the driving task, indirect modification of user behavior, and modification of exposure and route choice. This framework was chosen as it aligns well with our research questions. Next, the sub-categories were developed following principles of inductive category development by Mayring (2000). This approach was data-driven and emergent based on the semantic content of the interview data. Common steps of text analysis were applied to scrutinize the interview transcripts line-by-line, such as underlining/highlighting the text, writing notes, searching for keywords, and jumping between text passages (Mayring, 2000). Raw quotes were used to develop the sub-categories. The assignment of the quotes to sub-categories was based on repetition, similarities, and differences. This was an iterative process, which involved the re-assessment of the assignment of the quotes to their sub-categories during the coding phase. This emergent nature required the use of a single coder (Smith and McGannon, 2018), supporting the interpretative nature of qualitative research (O'Connor and Joffe, 2020). A single coder was used in qualitative emergent interview research before (Berge et al., 2022). The authors discussed the assignment of the quotes to their underlying sub-categories.
3. In the third step, the number of sub-categories mentioned by respondents was counted. When a sub-category was mentioned more than once per respondent, the number of mentions equaled a frequency of 1. Sub-categories had to be mentioned by at least five respondents to be eligible for the formation of a sub-category. When a quote was assigned to more than one sub-category, within each sub-category, this quote received a frequency of 1.
4. In the fourth step of the analysis, a maximum number of five illustrative quotes were selected to portray the meaning of each sub-category. Multiple mentions of a sub-category per respondent were not discarded but merged with the other mentions of the sub-category by the respondent. Therefore, some of the quotes represent clusters of sentences mentioned by the same respondents at different points during the interview. Filler words and repetitions (e.g., “you know,” “like,” and “uhhm”) were removed from the quotes.

## 3. Results

A total of 103 semi-structured interviews were conducted between February and June 2022. On average, an interview lasted 01:18:05 hours. Respondents were, on average 43 years old, with a standard deviation of 14 years; 91% were men, and 9% were women. Notably, 52% had a bachelor's or master's degree, 27% a college degree, 13% a high school diploma, and 8% a PhD degree. The three most common residential locations were California (20%), Colorado (8%), and Florida (7%). Respondents were reported to be engineers (30%), managers (8%), or retired (7%); 82% of respondents indicated having access to FSD Beta and standard Autopilot, while 18% had access to Autopilot. Respondents' average use of Autopilot and FSD Beta was 26.8 and 8.14 months, respectively. Respondents' frequency of the use of Autopilot and FSD Beta was 4.11 and 4.50, respectively (measured on a scale from 1 = less than monthly, 2 = less than weekly but more than once a month, 3 = 1–2 times a week, 4 = 3–4 times a week to 5 = at least five times a week). Five interviews were conducted in German (R04, R09, R15, R47, and R89) as these respondents were German native speakers.

The results of the data analysis are divided into three main categories and 15 sub-categories.

**Figure 1:**
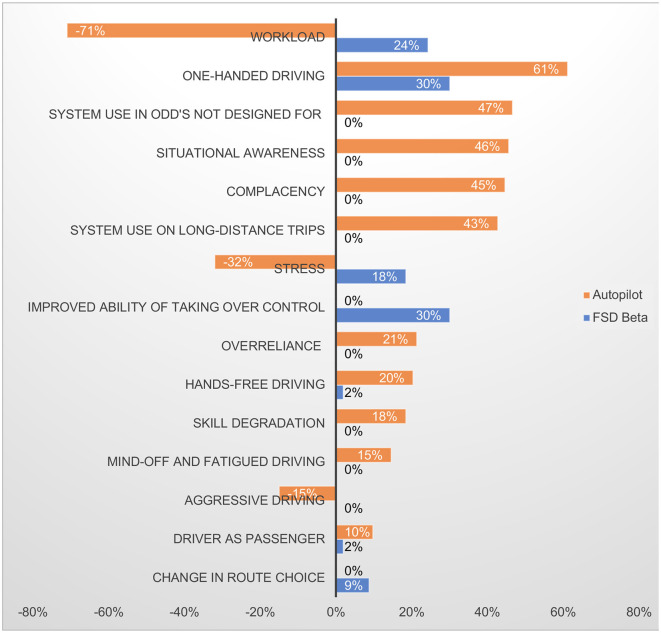
Visual representation of results of data analysis, sub-categories extracted from the interview data; positive values denote an *increase*, and negative values a *decrease* in the occurrence of driver state and behavior captured by the sub-categories.

The three main categories are direct, short-term behavioral adaptations (3), indirect, long-term behavioral adaptations (10), and changes in travel behavior (2). Figure 1 presents an overview of the main sub-categories. The three sub-categories mentioned most often pertained to the decrease in workload, an increase in one-handed driving with Autopilot engaged, and using Autopilot in ODD's not designed for.

Table 2 presents an overview of the main categories and sub-categories extracted from the data analysis, their expected effect, and the meaning of the sub-categories. Expected effects (column 3 in Table A1) were highly consistent over participants in most cases (+ or –), while unclear results were found in other cases (*NA*). The results are discussed in the subsequent sections.

**Table 2:** Results of data analysis, i.e., main category, sub-category, expected effect (negative = –, positive = +, *NA* = not available), the meaning of sub-category, and number (*n*) of respondents mentioning sub-category (total, Autopilot, FSD Beta).

**Main category**	**Sub-category**	**Expected effect**	**Meaning**	***n***		
				**Total**	**Autopilot**	**FSD Beta**
Direct, short-term behavioral adaptations	Situational awareness	+	Increase in situational awareness, being more attentive to vehicle's operation and surroundings	47	47	–
	Workload	–	Decrease in mental and physical workload with Autopilot engaged due to lateral and longitudinal control of driving task, and increases in situational awareness, resulting in reduction of driver fatigue, making driving and engaging in small secondary activities (more) relaxing and easier compared to manual driving	73	73	–
		+	Increase in workload with FSD Beta engaged given status as technology under development and high disengagement rate, making driving not relaxing	25	–	25
	Stress	–	Decrease in stress with Autopilot engaged due to lateral and longitudinal control of driving task, reduction in workload, and aggressive driving (including speeding and reckless driving)	33	33	–
		+	Increase in stress while driving with FSD Beta engaged given status as technology under development and constant need for supervision	19	–	19
Indirect, long-term behavioral adaptations	Aggressive driving	–	Decrease in driver aggression toward other drivers on road (including speeding and reckless driving) with Autopilot overtaking driving in stressful and monotonous situations and interacting with other road users	15	15	–
	Driver as passenger	*NA*	Driver becoming passenger with Autopilot and FSD Beta engaged due more monitoring rather than actively driving vehicle	12	10	2
	Overreliance	+	Overreliance on Autopilot due to expected safety benefits when driver is distracted, impaired or during inclement weather conditions (e.g., rain, night), and overtrust in system capability proven over time	22	22	–
	System use in ODD's not designed for	*NA*	Using Autopilot in ODD's for which it was not designed (i.e., non-highway roads, roundabouts, curves, hills, unmarked roads, poor visibility conditions) (Tesla, 2022a) due to lack of knowledge, experimental attitude to test the system, overtrust, or apparent system competence	48	48	–
	Complacency (eyes-off road driving)	+	Increase in risk of complacency (eyes-off road driving) with Autopilot engaged due to consistently solid system performance proven over time, overtrust in system capability, contributing to failures to monitor automated driving system, and engaging in secondary tasks	46	46	–
	One-handed driving	+	Increase in one-handed driving due to more comfortable driving experience, overtrust in system capability proven over time, and the need to satisfy the steering wheel torque sensor	63	63	31
	Hands-free driving	+	Increase in hands-free driving beyond the technical system specifications (i.e., nagging), enabled by e.g., weighting the steering wheel, or placing knees on steering wheel, resulting in complacency and secondary task engagement	21	21	2
	Mind-off and fatigued driving	+	Increase in mind-off and fatigued driving with Autopilot engaged (i.e., mind wandering, sleeping) due to overtrust in system capability proven over time	15	15	–
	Ability of taking over control	*NA*	Improved ability of taking over control with FSD Beta engaged given status as technology under development and constant need for supervision in comparison with impaired ability of taking over control with Autopilot engaged, with more relaxed placement of eyes, hands, and feet in familiar, trustworthy situations	31	–	31
	Skill degradation	+	Loss of skills, and increase of discomfort and loss of control driving traditional, non-automated car	19	19	–
Changes of travel behavior	Amount of traveling	+	Using Autopilot on long-distance road trips due to efficiency and safety benefits and positive effects pertaining to driver state	44	44	–
	Route choice	*NA*	Changes in route choice with FSD Beta engaged given its status as technology under development and motivation to contribute to the development of system and fully automated driving	9	–	9

The total number of mentions of the sub-categories excludes double mentions by the same respondent across n for Autopilot and FSD Beta.

### 3.1. Research question 1: What are driver's direct short-term behavioral adaptations of using Autopilot and FSD Beta?

#### 3.1.1. Situational awareness

**Autopilot**

This sub-theme covers the changes in drivers' situational awareness in comparison to manual driving. Respondents mentioned an increase in situational awareness associated with using Autopilot, as the eyes could be placed on the operation and surroundings of the vehicle (e.g., other drivers and lanes).

“*I would even argue that it allows you to pay attention with more detail to what's happening around you, to watch the other drivers, and actually monitor the other lanes a little bit more. So, it appears that you have a situational awareness that I don't believe you have when driving on your own.”* (R001)

“*You can set more of your attention to other vehicles and lane lanes rather than the actual driving software. So, what is it doing with the steering wheel? What is it doing with the brake? It is actually quite a massive experience. You can actually use your attention for other things like that because you can see if someone is passing you too closely, or if you are passing a large truck, you can see if it is too close, you need to move over. If you are focusing on driving, you might not be able to use attention in those ways.”* (R002)

“*Actually, I spend a lot of the time looking at other drivers during Autopilot, and counting how many people are on their cell phones. It's about 1 in 10 that are actively using it, and texting as they drive.”* (R006)

“*This one's more geared toward Autopilot. Being more aware and more alert for long drives. So just kind of like Autopilot with airplanes, you have a little more freedom to be looking around and checking your environment, your surroundings.”* (R046)

“*Number one benefit is not having to just pay attention as much to the small fine motor movements, sticking into the center of your lane. You get to pay attention to the nearby traffic, pay attention to the cars around me much more. It frees up my capacity, pay attention to others.”* (R065)

Drivers' situational awareness might also drop due to an overreliance on the system; the dual role of the driver transitioning to being a passenger with the systems engaged, complacency, hands-free, mind-off, and fatigued driving, as will be shown below.

#### 3.1.2. Workload

**Autopilot**

This sub-theme covers changes in mental and physical workload, with Autopilot contributing to a reduction in workload and driver fatigue. Using the system made driving easier and more relaxing as drivers were no longer required to perform most of the tactical and operational parts of the driving task and to monitor the driving task permanently.

“*Autopilot took a lot of extra load off the driver. You basically didn't have to process as much, and weren't as mentally strained to do long drives.”* (R041)

“*Why do I use Autopilot on the highway? Just because it's a lot easier, a lot less brain work. I'm a computer programmer. I use my brain all day. I don't want to use it on the highway all day. I'd like a break.”* (R045)

“*Autopilot will deviate driver fatigue. The largest thing I noticed is just took so much of the mental workload of driving off my brain. I didn't need to worry about what speed I was going, about how fast the car is ahead of me were going, about maintaining my lane anymore. Those are all mental tasks that are part of driving that I no longer need to worry about.”* (R054)

“*The base Autopilot is very helpful on the Interstate for reducing driver fatigue. It's a mental stress reliever because you don't have to do the micro corrections, it's doing that for you.”* (R090)

**FSD Beta**

FSD Beta, on the other hand, increased mental workload given its status as a developing technology and the corresponding need to constantly supervise the system as it could potentially do the wrong thing at the worst time. Respondents had to monitor the system and be ready to intervene and take over control anytime, resulting in a high disengagement rate.

“*FSD Beta, my disengagement rate is probably four or five disengagements per mile. Yesterday, I went to work and back and for 11-mile round trip, it was 43 disengagements. There's barely 43 intersections in my drive. As Autopilot tries to take over more tasks, it can actually be more work for me. It was just a straight road.”* (R007)

“*I use FSD Beta where I know I don't really need to give it a lot of energy. FSD Beta takes a lot of energy. Imagine having a 12-year-old on your lap. ‘Get off my lap. I don't have the energy for you right now.' It's doing certain turns, and then it gets confused or stuck. ‘Yes, it's OK to go ahead here, little child.”'* (R012)

“*FSD Beta – it's certainly very different in the way that I engage with it because I'm using it not as a relaxing thing as I would Autopilot. I'm using it more like a job. It could do the wrong thing at the wrong time. I really just have to keep an eye on. I have to kinda hover over it.”* (R048)

“*With FSD Beta, I see a lot of disengagement on the trip, I just disengage it and drive away, because at some point it becomes dangerous, I think, and it's easier for me.”* (R058)

“*Yes, I'm constantly like clicking the gas and pellet pushing the gas in the pellet to keep going. It's just a lot of work. It made no sense to use.”* (R090)

#### 3.1.3. Stress

This sub-theme covers differences in stress while driving when Autopilot and FSD Beta are engaged.

**Autopilot**

Autopilot contributed to a decrease in stress while driving due to its consistently reliable performance of keeping the vehicle within its lane and maintaining a safe distance to the car in front compared to manual driving, allowing drivers to engage in smaller secondary activities.

“*I would turn on Autopilot, and it would take all of the stress away. I didn't have to worry. It was smoothly keeping up with traffic and do well.”* (R018)

“*Why do you use Autopilot? On the highway, I use it because it takes the stress out of normal driving up. When I drive on the Interstate with my very old car, stop and go traffic can be very stressful. You can even feel the adrenaline go up through your arm if you miss something and all of a sudden you get startled. I don't ever get startled with the Tesla driving with Autopilot on the highway.”* (R031)

“*So, I do use Autopilot frequently. I do feel less stressed when it's operating, I know it's gonna stay in my lane. It's not gonna hit the car in front of me. So, I can then get on the touch screen and find the station I wanna listen to or other things I probably shouldn't be doing while driving.”* (R071)

**FSD Beta**

FSD Beta, on the other hand, increased the stress while driving due to its technological state as Beta technology and the corresponding need to constantly supervise the system. Respondents described the system as a “drunk toddler,” “teenage student driver,” and “babysitting” FSD Beta.

“*Autopilot allows me to relax. FSD not so much. It's more like I'm a babysitter a little bit.”* (R027)

“*If I'm on a casual drive and I don't want to think, I will drive manually. With Full-Self driving you have to think more as a Beta tester because it's not done, so you have to be ready to take over. So, it adds an extra level of stress, and you have to be more attentive. You have to pay a lot more attention. So, because of that, I do take breaks from using FSD Beta.”* (R036)

“*Just like Autopilot in an aircraft. It lets you relax a little bit so now you're not having to directly fly the plane, but you still monitor. With Beta typically, when I turn it on, it adds more stress.”* (R096)

### 3.2. Research question 2: What are driver's indirect long-term behavioral adaptations of using Autopilot and FSD Beta?

#### 3.2.1. Aggressive driving

**Autopilot**

This sub-theme covered the reduction in aggressive driving toward other drivers on the road, including speeding and reckless driving when Autopilot is engaged, reducing the stress of driving, and making driving more relaxing and safer.

“*It removes a lot of the stress of driving. I just put it into Autopilot and if the cars are slowing down, speeding up, I don't really care. I just sit there and watch the road, whereas if I'm driving, I'm constantly trying to move around cars, and hitting the brakes and it's much more stressful. So, it makes driving a lot more relaxing.”* (R046)

“*I used to speed, and I used to drive pretty dangerously. With Autopilot, I just don't worry about it anymore. I'll get to my destination when I get there, and I just let the car handle the drive. So, it made me a safer driver. Autopilot just calmed me right the hell down. Just chill, driver. That's what I am now.”* (R054)

“*Another reason I really like using full self-driving is it is making me a better driver. When I outsource my driving to the car, the chances of me getting a speeding ticket go down by orders of magnitude. When there are other cars on the road that are weaving in and out of lanes and trying to get ahead of other drivers, I just don't really care. The human nature aspect of driving, the competitive nature, or the aggression that you might have toward another driver or if you've had a stressful day, none of those things really matter anymore.”* (R065)

“*I'm coming from a place where I have multiple speeding tickets. I was … I'm a very aggressive driver. I have reckless driving charges. Autopilot probably just makes me a better driver because I just don't care anymore about speeding, about the aggression that other people are dishing out at me. It turns me into a very meek driver, it takes away all my aggression, basically.”* (R074)

#### 3.2.2. Driver as passenger

**Autopilot and FSD Beta**

This sub-theme covers the role of drivers as passengers with Autopilot and FSD Beta engaged, with respondents mentioning that they mainly monitor the operation of the system as passengers while still being required to take over control and operate the car as drivers.

“*I'll be completely honest with you. When I first got it [Autopilot], I probably wasn't paying … I probably was a little bit over trusting maybe. I ended up hitting road debris or something, and then it kind of scared me and I was like ‘Oh, crap. I actually have to pay attention.' So that first experience probably jolted me back into to like ‘I'm not a passenger.”'* (R006)

“*I described the FSD as a 5-year-old learning to drive right and then inflicting that on you as a passenger.”* (R042)

“*The benefit just in general is, it feels a lot more like being an observer. Almost being in a car with a student driver or being in a car with a teenager where you're kind of just observing them, you're not really doing anything for them.”* (R045)

“*When going through high traffic scenarios, I just let it maintain the distance between me and the car ahead of me and I just do my thing. I am a passenger in the vehicle, and I just need to make sure I don't miss my exit, or I don't miss my turn at that point.”* (R054)

“*I almost feel like I'm a passenger in the car, but it's like better than being a passenger because I'm still in control.”* (R068)

#### 3.2.3. Overreliance

**Autopilot**

This sub-theme covered respondents' (over-)reliance on Autopilot due to the expected safety benefits in situations in which drivers are distracted, impaired, during inclement weather conditions, or want to relax from driving, engaging in small secondary activities (e.g., selecting music and having a phone call).

“*If I'm tired or if I'm not paying enough attention, just knowing that it's going to be able to drive for me for a short amount of time is what it does best. Especially on the highway, I know that I can just kind of not go to sleep or anything, but I can relax and lean back and just give myself a mental break for a few minutes and I know that the car is gonna handle everything without any issues.”* (R022)

“*If I want to have that second set of eyes kind of patiently paying attention to the road where I know that maybe I'll be distracted by selecting music or on a phone call or honestly, if I've had a few too many drinks, it really does come in handy for that.”* (R027)

“*There's plenty drives where you are tired, at the end of the workday. Sometimes as a driver, you're not paying perfect attention, you're not fully energized, you're tired, you're distracted. It just feels like a safety thing too.”* (R070)

“*I actually do feel safer on that on limited access highways at night when I'm very tired because driving without such assist, I'm afraid of microsleep events if I'm very tired or fatigued which is where you basically fall asleep for maybe one or two seconds. Having a system there to back me up in those sort of situations makes me feel significantly safer than driving a standard vehicle. This came into effect two weeks ago for me.”* (R090)

#### 3.2.4. System use in ODD's not designed for

**Autopilot**

This sub-theme covers using Autopilot in ODDs for which it was not designed due to the apparent system competence to operate in these situations, a lack of knowledge, (over-)trust, or curiosity to use it in these situations.

“*I used it from the start, and then noticed very quickly that I can use Autopilot also on roads other than freeways.”* (R039)

“*The first time I used Autopilot, I didn*'*t really realize what it was meant for. It was meant for highway driving. I was using it on streets.”* (R052)

“*There was one instance where I was crossing an intersection and Autopilot just braked out of nowhere and I was really confused and all of a sudden, a car in front of me passed through at a high-speed flying by, when I had a green light.”* (R075)

“*Autopilot – that's what traditionally was only on the freeway, but you could use Autopilot in town, and I use it all the time. You've been in town even though it didn't work very good in town.”* (R095)

#### 3.2.5. Skill degradation

**Autopilot**

This sub-theme covers the loss of driving skills associated with using Autopilot, with respondents reporting feelings of discomfort, fear, and loss of control driving manually because they have been used to driving with the system automating a part of the driving task.

“*I would be very uncomfortable taking any long-distance road trip without Autopilot. I probably wouldn't take it in a car unless I absolutely had to.”* (R012)

“*A few months ago, I drove a friend's car and it was just a regular car. It was a road trip and I just remember being so focused on the road and I felt like I couldn't even turn my eyes away from the road because it was so scary.”* (R027)

“*It's really hard for me to get into the traditional car. I feel really safe in a Tesla. Whenever I'm going in the mountains here in Colorado, not ever having to touch the brake pedal when you're going down a hill and letting regenerative braking slow you down and doing so in a very controlled manner, makes me feel very safe, but most importantly in control. The way I feel when I'm in a traditional car and I'm coasting because I'm going downhill or coasting and having to brake, I feel extremely out of control of the car.”* (R065)

“*As soon as I have to take over, I feel very uncomfortable for some reason. I used to be very comfortable driving on my own.”* (R074)

### 3.3. Research question 3: How do drivers place their eyes, hands, and feet when using Autopilot and FSD Beta?

#### 3.3.1. Complacency (eyes-off-road driving)

**Autopilot**

This sub-theme covers the risk of complacency associated with using Autopilot. Complacency implies the failure to monitor the system due to consistently reliable system capability proven over time in simple, trustworthy environments, lulling drivers into a false sense of safety. It can result in taking eyes off the road and engaging in secondary eyes-off-road activities, such as reading and working on an electronic device or laptop.

“*With Autopilot, I pretty much don't pay that much attention anymore on roads that I'm familiar with. If my boss or someone sends an email, I am more comfortable, taking my eyes off the road for 15–20 seconds to reply. I look up occasionally just to make sure there's something on the road. Usually, I am reading something, flipping through a presentation or emails, reading paper materials or something on my phone or iPad. I've actually always done that even without a Tesla. I used to read an entire Wall Street Journal on my phone in a Honda Civic.”* (R033)

“*I have gone 44 miles without looking up on Autopilot. It's all on freeway, but I don't even have to worry about it. So that part is great. So it's not a difficult, complicated drive, but I don't worry at all about it.”* (R042)

“*This is an experience I have on Autopilot I've had a giant block of Styrofoam in the road and of course, I wasn't attentive. I've drove this road over so often. We're not paying attention. I talked with Polly about something, and I look ahead and in front of me is that piece of Styrofoam. The car didn't see it, and we ran it over.”* (R048)

“*When Autopilot is active, I feel safe enough that I can take my eyes off the road for a couple minutes and I generally will only do that if I can see really far in front of me, I'm going straight and there's no turns. Then I know it's safe to take my eyes off the road. I know you shouldn't do that, but I'm doing it in a controlled environment where I've had almost three years of experience with Autopilot.”* (R055)

“*I did have one experience where I used Autopilot along a straight country road, no one around, just like cows or whatever, in the middle of nowhere, and I decided to pull out a laptop, and worked on a laptop while the car drove, 45 miles an hour or something like that, and it was going great until the car came into a small town where the speed limit dropped by 25 miles an hour and I totally didn't notice and got pulled over instantly by the local town cop.”* (R092)

**FSD Beta**

Respondents were more prone to monitor the road with FSD Beta, which required permanent supervision by drivers for the reasons mentioned before.

“*Talking about the Beta that I have for city driving, you need to be 150% paying attention because the car is going to do something stupid. That thing should not be released to the masses now. It really should not. I hope they don't.”* (R001)

“*FSD Beta. I can't look away even for a half second. It just changed lanes on its own.”* (R007)

“*I feel like if I didn't intervene right there, that truck that was stopped at that light would have gotten hit and that would have been a collision. That's one good example of FSD Beta why I need to stay active. I need to pretty much be alert at all times right there to take over because it will do the wrong thing at the wrong time.”* (R048)

“*Generally testing FSD Beta, I know that I'm using software that is not ready for any random person in the world to use because it requires literally full attention all the time. I'm driving at like 200% attention with FSD Beta. For Autopilot, it's like 80% of my normal attention while driving. There's just not much to pay attention to when it goes straight.”* (R055)

#### 3.3.2. Mind-off and fatigued driving

**Autopilot**

This sub-theme covers instances of mind-off and fatigued driving associated with using Autopilot in comparison to manual driving, with respondents reporting engaging in mind wandering to nodding off behind the wheel. Respondents were prone to fall asleep on long-distance drives as the result of overtrusting the capability of the system proven over time, negligence, or necessity to drive even though their mental capabilities did not match the demands imposed by the driving task.

“*When I was driving home a week or two ago from Maryland, it was one of those cases where you get on the highway and your next offramp is in 200 kilometers. You get into the groove; you're listening to music. You're not paying attention. Suddenly, my car put its blinkers on and started to change into the offramp and I'm like ‘What? What are you doing?' I disengaged Full Self-Driving thinking it was a problem, and then I looked at the map and I'm like ‘Oh, this is my offramp.' I would have missed my offramp if it wasn't for FSD.”* (R044)

“*After 17 or 18 hours of driving, I just did the little nod where it happened very quickly. I know I was completely awake again and I was like ‘OK, well, here's the time where I have to take the weight off.”'* (R068)

“*I can't say I haven't nodded off with Autopilot engaged. I've have driven a lot while being tired over the years from doing side work and there are times where I'm used to that nodding off at the wheel. My brain tends to jerk myself awake. Usually, my hands are on the wheel, so they are giving the car feedback. So the car itself is not aware that I have nodded off.”* (R071)

“*When you have Autopilot, it's your subconscious driving while you can think of other stuff. Your brain is focused differently on driving while being safe. You're driving and then you come out of a thought and you're like ‘How did I get here? I don't remember driving this part'. With Autopilot, it's driving like that all the time because you're able to think of other stuff without being fully concentrated on driving.”* (R073)

“*I've actually nodded off using FSD on long highway drives. In those regards, I'm confident, but I would never do that when there's cars around. Where we live, you can literally see 10 kilometers down the road and there's no car. It's just straight. That's when I could relax, and I was impressed. I was a little embarrassed to tell people that I had done that because people raised their eyebrows ‘You did what?' If I saw any cars, no, I was paying attention.”* (R081)

#### 3.3.3. Position of the hands-on steering wheel

**Autopilot and FSD Beta**

**Figure 2:**
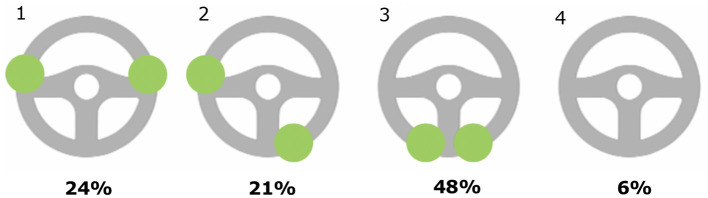
Placement of hands-on steering wheel with Autopilot engaged. Multiple responses were allowed.

**Figure 3:**
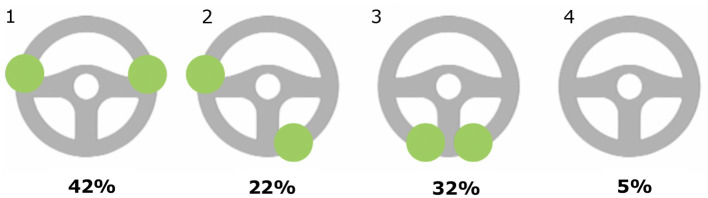
Placement of hands-on steering wheel with FSD Beta engaged. Multiple responses were allowed.

This sub-theme covered the position of respondents' hands on the steering wheel when Autopilot and FSD Beta are engaged, as shown in Figures 2, 3 that resulted from respondents being asked to report how they placed their hands on the steering wheel. As shown in Figure 2, when Autopilot is engaged, 48% of respondents reported placing their hands at the bottom of the steering wheel, followed by the “3–9 o'clock” position. As shown in Figure 3, when FSD Beta is engaged, 42% of respondents reported having their hands at the “3–9 o'clock” position, followed by 32% of respondents placing their hands at the bottom of the wheel.

**Figure 4:**
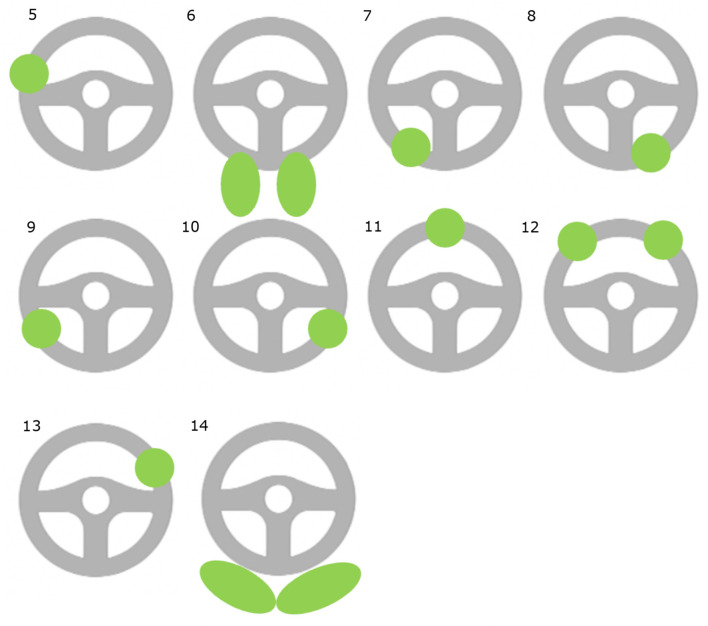
Additional positions of hands and feet with Autopilot and FSD Beta engaged.

In this process, respondents mentioned additional positions of their hands and feet when Autopilot and FSD Beta are engaged. As shown in images 6 and 14 of Figure 4, respondents mentioned situations in which they drove with their knees placed on the steering wheel and legs crossed, respectively, when Autopilot was engaged.

##### 3.3.3.1. One-handed driving

**Autopilot and FSD Beta**

Respondents mentioned that they engaged in one-handed driving with both systems engaged. Driving one-handed was considered more comfortable or occurred to satisfy the steering wheel torque sensor. Respondents questioned the suitability of the torque-based steering wheel driver monitoring system as not being necessarily effective in driver monitoring and guaranteeing safe use. It was mentioned that rather than actively steering the vehicle, the hands were placed on the steering wheel mainly to satisfy the steering wheel torque sensor. The weight of two hands placed on the steering wheel could result in accidentally disengaging the system, contributing to a decrease in the driver's perceived safety.

“*The hands-on feature is a very clumsy method in my opinion of ensuring driver attention. I'd much rather be on the road with someone whose hands are off the steering wheel, but they're paying attention than someone who's hand is resting on the steering wheel, but they're checking their email.”* (R007)

“*I keep a hand on the steering wheel at all times. Not both hands. I keep the hand on just enough to satisfy the torquing requirement where there needs to be weight on the system.”* (R054)

“*They could get off the steering wheel nag because actually you could apply too much torque while you're trying to make sure it knows you're paying attention and that takes it out of Full Self-Driving Beta, and that could be a shock if you aren't ready for it. My hands are always on the steering wheel but to make sure you have to keep the torque all the time - it's a safety issue.”* (R062)

“*I think probably still one-handed. If I'm using two hands, I'm just driving myself usually. It feels kind of hard using two hands while using Autopilot because the torque thing, and you don't wanna touch it enough to take it out of it.”* (R070)

##### 3.3.3.2. Hands-free driving

**Autopilot**

This sub-theme covers incidences of hands-free driving with Autopilot engaged, with respondents removing their hands from the steering wheel longer than is technically allowed by the system. Hands-free driving for long stretches of time represents a knowing violation of the system as it was enabled by placing weights on the steering wheel to satisfy the steering wheel torque requirement.

“*It is hands off for me*.^1^ *I have work arounds for everything where I don't need to pay attention as much.”* (R033)

“*You put a one- or two-pound weight under the steering wheel. I use that all the time. So, I have a tungsten bar that's really heavy metal and then I attach that with Velcro to the steering wheel, and it thinks my hand are always attached.”* (R044)

“*Honestly, this is what I do most of the time. I'll actually have a little weight that you can clip on to the steering wheel that negates all the nagging that it does.”* (R068)

“*Autopilot – I rarely have my hands on the wheel because when I turn it on, I know that it's safe and I know I don't really need to worry too.”* (R077)

“*I have a little coin pursue three stacks of 50 pennies started 50 US heavies. I found the perfect amount of weight and it's not gonna snap it out, but it's going to keep it in a lot of power. And it's small enough that I can grab it with my hand. I only really use that on the highway.”* (R094)

**FSD Beta**

FSD Beta, on the other hand, reduced the likelihood of hands-free driving given its status as technology under development and the corresponding need to constantly supervise the system.

“*I'm hyper attentive of it. My hand isn't just like on Autopilot, loosely hanging on the wheel. With FSD Beta, my hand is gripping the wheel, ready to when it tries to turn left or something.”* (R045)

“*FSD Beta – I even have a separate grip for the wheel then when I'm on Autopilot. Both hands on the wheel, I might have to apply immediate torque quickly.”* (R063)

“*My hands are on the wheel a lot more than Autopilot is. They're always on the wheel. Eyes are always on the road. I don't like looking away, even for a second.”* (R066)

“*I've seen a video recently of someone in Canada making videos and his hands are in his lap. Clearly, he hasn't experienced the things that I've experienced because if he had, you wouldn't do that. You understand that you don't have the time to not have your hands on the wheel. Sometimes things will happen very quickly.”* (R096)

#### 3.3.4. Ability to take over control

**Autopilot and FSD Beta**

This sub-section covers drivers' ability to take over control with Autopilot and FSD Beta engaged. Respondents mentioned that they were more likely to stay prepared to take corrective actions with FSD Beta, reporting a heightened sense of awareness and readiness to take over control in terms of a more tense placement of eyes, hands, and feet. They reported being less prepared to take over control with Autopilot engaged due to a high level of trust in the capabilities of the system proven over time.

“*For Autopilot, I'd be guilty of not being prepared to take corrective action 100% of the time. A big part of the reason I love Autopilot is I don't have to, but for FSD Beta I would say ‘Yes, I am prepared to take corrective action at all times'.”* (R012)

“*My hand is on the wheel. My foot is on the floor. I am watching the road. I can take corrective action at any moment but there's a very different state of mind from Full Self-Driving ready to take corrective action. I am almost sitting there tense, my hand is maybe at the same place for Autopilot, but with Full Self Driving, I'm gripping the steering wheel, it's ready to go. I have a foot over one pedal or the other, depending on whether I think it's going to be from too slow or too fast.”* (R018)

“*I certainly do that on FSD Beta. I definitely do for all the reasons I've said before. But Autopilot – I feel like I'm typically less prepared because I'm more relaxed. I'm more letting my guard down because I trust it more. It's never done as much wrong as it has on FSD Beta. I'm looking at the scenery, I'm enjoying the ride. I'm enjoying the ride versus driving pretty much. So definitely less prepared on Autopilot.”* (R048)

“*I typically stay prepared to take corrective actions. So, with Autopilot, not necessarily because if vehicles are braking, it's really good at predicting the braking as needed. Lane changes – it takes 30 seconds for it to make a lane change, so it's more of the system knows when it's able to do so safely, so I just let it do it. Obviously, Beta. You always have to be ready to take over at any time. Again, my feet and hands are always ready to take over, so with FSD always usually prepared to take over.”* (R075)

“*Autopilot – I would not see I'm prepared to take corrective action because there's not a huge corrective action elicited requirement. Full Self-Driving Beta – your hands are on the wheel, your foot's over the brake, your hands over the stock. You have all three ways to murder it to turn it off at your disposal, and you're actively ready to do so.”* (R084)

#### 3.3.5. Placement of feet

**Autopilot and FSD Beta**

The placement of feet when Autopilot is engaged was reported to be more relaxed compared to FSD Beta, placing the foot flat on the ground or taking the shoes off and sitting cross-legged on the driver's seat. With both systems engaged, respondents mentioned that they placed their right foot in a hovering position over the accelerator rather than on the brake to be able to react to unexpected system behavior, such as phantom braking.

“*It's pulled back like whatever is most comfortable on those long highway rides. It's definitely not always over the brake pedal. For FSD Beta, you actually have to use the accelerator often. You can use the accelerator, move it along. If not, my foot is over the brake. So a lot of times my foot is so the whole time using FSD Beta.”* (R002)

“*FSB Beta – I'll keep my foot right above the accelerator, ready to hit the gas or the brake. Autopilot – I'll put my feet flat on the ground. I won't have them sitting on top of the accelerator on, I'll have him relax. If you would set cruise control, same thing.”* (R043)

“*Especially on long trips, I will often take my shoes off. If I'm driving on a long drive and it's a dual carriageway divided and it's nighttime, I know there's gonna be almost no chance of a phantom brake because there's no shadows and there's no oncoming traffic. I know, I shouldn't, but sometimes I'll sit cross-legged on the driver's seat.”* (R044)

“*Yeah, with Autopilot, I'm pretty relaxed. I got my left foot to the far-left side of the footwell, it's got a little foot rest built into the car right there. On Beta, my right foot is mostly hovering over the accelerator more than it's hovering over the brake because the car has phantom braking.”* (R048)

“*FSD Beta – I usually have my foot hovering over the accelerator because it is more likely to phantom brake or slow down than anything. Autopilot, If I'm on a freeway stretch for a long time and I've driven it many times before, I'll pull both my feet back into like a normal sitting position.”* (R055)

### 3.4. Research question 3: How does using Autopilot and FSD Beta affect driver's amount of traveling and route choice?

#### 3.4.1. Amount of traveling

**Autopilot**

This sub-theme covers the increase in the amount of traveling associated with using Autopilot, with respondents reporting engaging in long-distance drives as Autopilot contributed to a reduction of driver stress and fatigue and an increase in perceived safety.

“*It's definitely made drives that otherwise would have been impossible. I went to Texas by myself, 1400 miles each way. Doing that distance by myself over the course of like a day and a half, I don't think I could have done that in a car without driver assistance features and especially at the level that Autopilot provides.”* (R005)

“*We did this 3100-mile road trip to Florida. We did a 4500-mile road trip to Texas. We did an 1800-mile road trip to Harrison back and we've done a bunch of trips to the Hudson Valley and that's just in the last year and all of those trips were made possible because of Autopilot.”* (R012)

“*I also realized that when I get to my destination, I'm way less exhausted. The one of the drives I recently did, I was able to drive for 18 to 20 hours and arrive at the destination wide awake, right?”* (R054)

“*What I like about it, like for instance, driving a long trip. This summer, I'm gonna drive it from Phoenix out to Southern California, 66/67 hours.”* (R072)

“*So inherently I feel vastly safe with Autopilot. I feel 5X more safe driving so much so that I'm willing to make more road trips, and I'm willing to take longer road trips.”* (R086)

#### 3.4.2. Route choice

**FSD Beta**

This sub-theme covers changes in route choice associated with using FSD Beta. Given the Beta nature, respondents reported using the system to test it, e.g., in challenging and new situations, to contribute to the development of the system.

“*I'll just drive the majority of the time that I use FSD Beta to test something. I have a couple of standard routes that I do and then sometimes I'll use it if I'm in a new area, and I wanna see how it's gonna handle something.”* (R051)

“*I only use Beta like I did it this morning. It's Sunday morning 6:30. I was just plotting trips to see how it could handle, and how it did without any people around.”* (R068)

“*I just use FSD Beta to unwind in the evening. I have an app on my phone, and I have a couple 100 addresses in town, and it just randomly shuffles where it's gonna drive to, and so I literally just drive around town, and look for edge cases that I have to correct.”* (R085)

“*FSD Beta I pretty much only engage it on specific test routes that I know how the car has performed and I'm trying to do a comparison so I can get an idea of how it's functioning and what improvements I'm seeing. I pretty much do like planned routes where I can test situations with it.”* (R094)

## 4. Discussion

### 4.1. Synthesis of results

This study reports 103 in-depth interviews with drivers whose cars were equipped with Tesla's FSD Beta and standard Autopilot to investigate the driver's direct short-term and indirect long-term behavioral adaptations of using Autopilot and FSD Beta and changes in driver's travel behavior.

The data analysis from the interviews resulted in three main categories and 15 sub-categories. The three main categories mentioned by at least five of 103 respondents were driver's direct short-term behavioral adaptations of using Autopilot and FSD Beta (3), indirect long-term behavioral adaptations of using Autopilot and FSD Beta (10), and changes in the amount of traveling (1) and route choice (1).

The direct short-term behavioral adaptation of drivers (3) pertained to an increase in situational awareness with Autopilot engaged, a decrease in mental and physical workload and stress with Autopilot engaged, but an increase in workload and stress with FSD Beta engaged.

The indirect long-term behavioral adaptation of drivers (10) was a reduction in aggressive driving with Autopilot engaged; the dual role of the driver transitioning to being a passenger in the car; overreliance on Autopilot; using Autopilot in ODD's for which it was not designed; complacency (eyes-off-road driving); one-handed driving, hands-free driving; mind-off and fatigued driving; an improved ability to take over control with FSD Beta engaged; and skill degradation resulting in drivers being uncomfortable without using automation.

The changes in the amount of traveling (1) pertained to more (very) long-distance trips with Autopilot and route choice with FSD Beta engaged (1).

The three sub-categories mentioned most often were a decrease in workload with Autopilot engaged, an increase in one-handed driving, and using Autopilot in ODD's not designed for. The sub-categories mentioned most frequently may represent the most significant and prevalent behaviors of human drivers in partially automated cars, some of which are already well-documented (e.g., reduced workload, see section 4.2.2 below). However, this does not necessarily mean that sub-categories mentioned less frequently are of minor importance for Human factors researchers and professionals (e.g., the sub-category “mind-off and fatigued driving” represents an undocumented yet highly safety-critical behavior).

### 4.2. Research question 1: What are driver's direct short-term behavioral adaptations of using Autopilot and FSD Beta?

#### 4.2.1. Situational awareness

Respondents reported an increase in situational awareness with Autopilot engaged in comparison to manual driving. They reported being able to monitor the vehicle surroundings more, which they considered a key advantage of using Autopilot compared to manual driving. This reflects studies showing that respondents identified observing the scenery or landscape as one of their favorite activities during automated driving (Pfleging et al., 2016; Nordhoff et al., 2021). Furthermore, this corresponds with studies reporting increased situational awareness when automated driving was engaged (De Winter et al., 2014; Endsley, 2017). However, the role of the driver transitioning to being a passenger with the systems engaged, cases of overreliance, complacency, hands-free, mind-off, and fatigued driving may diminish drivers' actual situational awareness, even when they report increased situational awareness.

Respondents reported placing their eyes more on the vehicle surroundings with Autopilot engaged and more on the road ahead with FSD Beta. More dispersed visual attention and longer times spent watching the driving scene have been associated with higher situational awareness during automated driving (Liang et al., 2021). The present study did not discriminate the three levels of situational awareness (i.e., perception of the environmental elements and events, comprehension of meaning, and projection of status in the future) (Endsley, 1995). A high situational awareness on three levels is essential to successfully take over control of the car in critical transitions (Zhou et al., 2021). We recommend future research to investigate all levels of situational awareness objectively over time in real driving conditions (Liang et al., 2021; Zhou et al., 2021).

#### 4.2.2. Workload

This study found a reduction in physical and mental workload when Autopilot was engaged, reducing driver fatigue and increasing relaxation due to Autopilot taking over tactical and operational parts of driving on highways. This corresponds with company reports (Tesla, 2022b) and ample scientific studies reporting a reduced objective workload (see a metastudy by De Winter et al., 2014; Heikoop et al., 2019) and subjective workload (only for automation-experienced drivers) (Stapel et al., 2019). This increased objective workload found in the study of Stapel et al. (2019) was associated with active monitoring and is somewhat comparable to the increased attention and workload now reported with FSD Beta. Respondents in the present study were early adopters with a strong technical or professional background and a high level of technology savviness, which may also explain the low subjective workload. Studies have shown that cognitive underload can be detrimental to drivers' take over performance (McWilliams and Ward, 2021). Hence, it is debatable whether the expected improved ability to take over, as reported by our respondents, is realistic. In comparison to Autopilot, FSD Beta increased workload as unfinished automated driving technology and the corresponding need to constantly supervise the system, being prepared to take over control anytime as the system may do the wrong thing at the worst time.

#### 4.2.3. Stress

Respondents reported a decrease in stress when Autopilot was engaged due to Autopilot taking over the lateral and longitudinal part of the driving task on highways. This reflects studies reporting a decrease in stress during partially automated driving (Heikoop et al., 2019; Hardman, 2021). FSD Beta, however, increased stress while driving. We recommend future studies to investigate to what extent two systems or ODD's imposing totally different cognitive demands on drivers affect drivers' ability to monitor automation and intervene in critical situations over time.

### 4.3. Research question 2: What are driver's indirect long-term behavioral adaptations of using Autopilot and FSD Beta?

The use of Autopilot and FSD Beta induced some indirect, unintended, and potentially dangerous behavioral changes in drivers. Some behaviors represent knowing violations of intended use (e.g., weighting the steering wheel, using Autopilot in ODD's not designed for), and others reflect misunderstanding or lack of experience (e.g., using Autopilot in ODD's not designed for).

#### 4.3.1. System use in ODD's not designed for

Respondents reported using Autopilot in ODDs for which it was not designed due to a lack of knowledge on the ODD's in which it can be used, overtrust, apparent system competence, or to experience and test the limits of the system. Previous research supported the use of Autopilot in ODD's for which it was not designed (Kim et al., 2021), which may represent a safety risk. Stapel et al. (2022) revealed that drivers of partially automated driving systems used ACC and LKA as intended (i.e., mostly on highways and less on urban roads). Educating drivers using the user manual may be insufficient as manuals are rarely consulted to gain information about system capabilities and limitations (Kim et al., 2021) and may be too technical for non-technical users. Driver training programs may need to be established (Kim et al., 2021), and system limitations can be more effectively communicated via human–machine interfaces (Capallera et al., 2019). However, as a correct understanding of system capabilities and limitations does not guarantee safe use, safe use may need to be “established” by design, i.e., punishing misuse by deactivating the system for a predefined period, e.g., *Autopilot Jail* (Reddit, 2022b).

### 4.4. Research question 3: How do drivers place their eyes, hands, and feet when using Autopilot and FSD Beta?

#### 4.4.1. Complacency (eyes-off-road driving)

The present study provides evidence for complacency with Autopilot but not with FSD Beta. Respondents indicated becoming complacent over time, taking their eyes off the road for relatively long stretches (e.g., 44 miles without looking up), and engaging in secondary activities (e.g., working on a laptop). This concurs with other studies in partially automated driving reporting drivers taking eyes off the road (Solís-Marcos et al., 2018; Morando et al., 2021) and engaging in secondary activities (Endsley, 2017; Banks et al., 2018; Wilson et al., 2020; Kim et al., 2021; Metz et al., 2021). An on-road study by Stapel et al. (2022) did not reveal substantial differences in driver attention between manual driving and partially automated driving. In line with Hardman (2021), the current study revealed that drivers of partially automated driving systems felt like passengers supervising rather than actively controlling the vehicle during automated driving. This may increase the likelihood of complacency and hamper the ability to take over control in safety-critical situations.

#### 4.4.2. One-handed, hands-free, mind-off, and fatigued driving

Our study found evidence of one-handed, hands-free, and mind-off driving. Studies have provided empirical evidence that users of Autopilot provide low or even no steering wheel control (Banks et al., 2018; Kim et al., 2021; Morando et al., 2021). Our study revealed that the sensitivity of Autopilot's torque steering wheel sensor might encourage one-handed driving, which represents a violation of recommended use according to Tesla's driver manual, requiring drivers to keep their hands on the steering wheel at all times (Tesla, 2022a). Our study further enriches the literature, providing evidence for actively manipulating the steering wheel control by weighting the steering wheel to indicate driver availability. Mind-off driving was also observed, with drivers reporting engaging in mind wandering or even falling asleep behind the steering wheel when Autopilot was engaged. Mind wandering is more likely to occur during monotonous (Eastwood et al., 2012), and easier or longer tasks (Smallwood and Schooler, 2015). Ahlström et al. (2021) found that partially automated driving had a small effect on fatigue during the daytime but increased the likelihood of increased sleepiness during the nighttime.

### 4.5. Research question 4: How does using Autopilot and FSD Beta affect driver's amount of traveling and route choice?

Using Autopilot has contributed to (very) long-distance traveling due to safety and convenience benefits, which concurs with studies conducted for partially automated driving (Hardman et al., 2022). An increase in vehicle miles traveled has also been found for conditionally automated driving (Lehtonen et al., 2022). New safety risks may arise when the number of hours traveling by car with the system engaged exceeds drivers' mental and physical capacities to perform the driving task safely, i.e., respond to objects and events in the environment and take over requests. Studies have shown that humans have difficulties in effectively monitoring automation for more than 30 min (Bainbridge, 1983). FSD Beta contributed to a change in route choice, given the interest and curiosity of its Beta users to test the system in challenging situations, helping Tesla to develop the system and realize the dream of a driverless future. An increase in the number of local trips due to Autopilot was evidenced before (Hardman, 2021). It is plausible that these effects are temporary and disappear with system maturity.

### 4.6. Study limitations and implications for future research

First, the data extracted from the present study reflect the subjective perceptions of drivers, which may not correspond to actual behavior, risk, and safety. Social desirability bias may be present, with respondents providing socially desirable responses, or responses that reveal their actual behavior when Autopilot and FSD Beta are engaged.

Second, face-to-face interviews may produce biased responses due to the personal contact between respondents and the interviewer, e.g., the tone of the questions asked and the facial expressions of the interviewer (Bowling, 2005). This bias was reduced by developing the interview protocol with a set of predefined questions, with respondents being able to steer through the questionnaire themselves during the Zoom interview with the limited intervention of the researcher.

Third, self-selection bias is present with regard to participation in the study, entry into the FSD Beta program, as well as the initial purchase decision that is alluded to entry into the FSD Beta program. FSD Beta participants represent an exclusive group selected by Tesla in two stages, with Tesla granting access to FSD Beta to pre-selected owners in the first stage (e.g., presidents of Tesla Owner clubs, YouTubers, and influencers) and to drivers with a high safety score in the second stage (Korosec, 2021; Lambert, 2022a,b). As a result, the interest in, enthusiasm for, outspokenness, and positivity of respondents as early adopters of partially automated driving technology is higher than in the general population. As early adopters, respondents may also be more willing to take risks to test the limits of the system (Agarwal et al., 1998). We recommend future research to replicate the study with a representative part of the population of drivers of partially automated driving systems to investigate to what extent the findings of the present study can be transferred to the general population.

Fourth, our study did not systematically investigate to what extent the reported behavior (e.g., weighting the steering wheel, using Autopilot in ODD's not designed for one-handed driving) represents knowing/deliberate or unintended violations (see Åberg and Warner, 2008) of the use of Autopilot and FSD Beta, why these behaviors occurred (e.g., due to a lack of misunderstanding or experience), and how they could be prevented. We recommend future research to determine the voluntary or involuntary nature of driver behavior in partially automated cars and investigate adequate measures and interventions to prevent these behaviors (e.g., knowledge and awareness campaigns, in-vehicle safety design, or infrastructural measures) (see Atombo et al., 2016).

Fifth, our study did not investigate driver understanding and acceptance of the steering wheel requirement when Autopilot and FSD Beta are engaged. It should be investigated to what extent the steering wheel requirement during partially automated driving represents an example of abuse of automation, which is defined as the implementation of automation without paying appropriate consideration to the effects on its users (see Lee, 2008).

Sixth, our study formulated the “expected effects” of using Autopilot and FSD Beta on driver state and behavior (see Table 2) based on the results of the data analysis. We did not investigate the statistical significance of these effects. The results obtained on driver behavior and the use of FSD Beta can offer novel insights into how drivers accommodate and use partially automated driving in complex urban environments.

## 5. Conclusion

This interview study with 103 participants of Tesla's Full Self-Driving Beta program, which extends the ODD of standard Autopilot to non-highway roads, revealed that the use of Autopilot and FSD Beta resulted in unintended positive and negative changes in user behavior. Drivers became complacent over time with Autopilot engaged. They failed to monitor the system and engaged in hands-free, mind-off, and fatigued driving, such as placing weights on the steering wheel and falling asleep behind the wheel with the systems engaged. The risk of complacency and unsafe behavior was high in simple highway environments. The effectiveness of the steering wheel torque sensor as driver monitoring technology was questioned. Testing unfinished automated driving technology may place substantial demands on drivers who might be unprepared to meet these demands. We recommend future research to investigate to what extent unintended negative behavioral changes increase the likelihood of being involved in a crash compared to manual driving.

## Data availability statement

As some of the data is part of future publications, the data will be made available upon request after publication of the data in consecutive studies. For further enquiries please contact SN, s.nordhoff@tudelft.nl.

## Ethics statement

The studies involving human participants were reviewed and approved by Human Research Ethics Committee of Delft University of Technology in the Netherlands. The patients/participants provided their written informed consent to participate in this study.

## Author contributions

SN: conceptualization, methodology, software, validation, formal analysis, investigation, resources, data curation, writing—original draft, writing—review and editing, visualization, supervision, and project administration. JL, SC, SB, MH, and RH: writing—review and editing. All authors contributed to the article and approved the submitted version.

## Footnotes

^1^Respondent was asked whether Autopilot is a hands-free feature.

## Appendix

**Table A1:** Overview of the interview instrument, i.e., question number and question.

**Question number**	**Question**
Q1	Do you have the Full Self-Driving Beta (FSD Beta) feature? (1 = Yes, 2 = No)
Q2	Before the first time of using Autopilot and FSD Beta, did you watch/read/listen to information on how to use it? (1 = Yes, 2 = No)
Q3	Please mention the type of information you consulted on how to use Autopilot and FSD Beta [website of Tesla (www.tesla.com), car dealer/sales point, online communities and forums, YouTube videos, newspapers and magazines, friends, family, colleagues, driver manual]
Q4	Please describe your experience with using Autopilot and FSD Beta and the benefits and risks associated with using it. Please explain your answer
Q5	Have your expectations of using Autopilot and FSD Beta been fulfilled? Why/why not?
Q6	Why do you use Autopilot and FSD Beta?
Q7	Did you ever stop using Autopilot and FSD Beta (for prolonged periods of time)?
**Next, we would like to explore your perceptions regarding four general statements about the operation of Autopilot and FSD Beta**	
Q8	The current Autopilot does make driving autonomous. Is that correct? (1 = Yes, 2 = No, 3 = I don't know)
Q9	There are no safety issues with Autopilot. Is that correct? (1 = Yes, 2 = No, 3 = I don't know)
Q10	Autopilot is a hands-on feature. Is that correct? (1 = Yes, 2 = No, 3 = I don't know)
Q11	Tesla FSD Beta is safer than a human. Is that correct? (1 = Yes, 2 = No, 3 = I don't know)
**With the next section, we would like to explore your perceptions of safety while using Autopilot and FSD Beta**.	
Q12	Do you feel safe when Autopilot and FSD Beta is active? Why/why not?
Q13	What/how do you feel when you feel safe/unsafe? Please explain
Q14	What is it about Autopilot and FSD Beta that is safe/unsafe? Please explain
Q15	Now please remember the situation/s in which you typically feel unsafe when Autopilot and FSD Beta is active and describe these situations
Q16	What can Autopilot and FSD Beta do to support your safety in Autopilot and FSD Beta? Please explain
Q17	Does feeling safe/feeling unsafe impact how you use Autopilot and FSD Beta on your next drives/in the future? Please explain
Q18	Has your perceived safety changed over time? If so, how?
**With the next section, we would like to explore your trust in Autopilot and FSD Beta**	
Q19	How would you position your level of trust in Autopilot and FSD Beta (1 = I don't trust it at all, 2 = I don't trust it, 3 = I neither don't trust it at all nor trust it a lot, 4 = I trust it, 5 = I trust it a lot)
Q20	What can Autopilot and FSD Beta do to support your trust in Autopilot and FSD Beta?
Q21	Does your trust/distrust in Autopilot and FSD Beta impact how you use Autopilot and FSD Beta on your next drives/in the future? Please explain
Q22	Has your trust changed over time? If so, how?
Q23	When you do compare yourself with other drivers, Autopilot, and FSD Beta, do you think you are ... (1 = A much worse driver, 2 = A worse driver, 3 = Not a better nor a worse driver, 4 = A better driver, 5 = A much better driver) (De Craen, 2010)
**With the next section, we would like to explore how you typically use Autopilot and FSD Beta**	
Q24	How do you typically place your hands on the steering wheel when Autopilot and FSD Beta is active? Please select the image that serves as best representation of your placement of your hands on the steering wheel when Autopilot/FSD Beta is active and explain your answer Figure is from Morando et al. (2021)

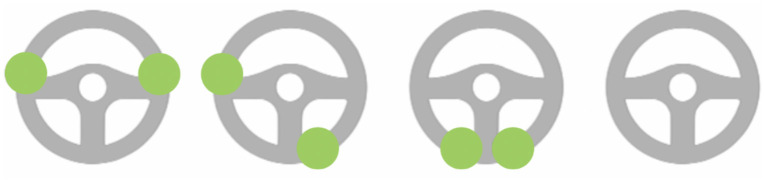
	
Q25	Do you typically keep your hands on the steering wheel at all times?
Q26	Are you typically fully attentive and alert at all times?
Q27	How often do you typically engage in other secondary activities while Autopilot and FSD Beta is active? (Never, rarely, occasionally, frequently, always; monitoring the road ahead, talking to fellow travelers, observing the landscape, using the phone for music selection, using the phone for navigation, using the phone for calls, eating and drinking, using the phone for texting, watching videos/TV shows, sleeping)
Q28	Do you disengage Autopilot and FSD Beta? Why/why not?
Q29	Does Autopilot and FSD Beta disengage? When/in which situations?
Q30	How do you typically place your eyes when Autopilot and FSD Beta is active?
Q31	Do you typically keep your eyes on the road at all times?
Q32	Do you typically monitor the vehicle and its surroundings at all times?
Q33	How do you typically place your feet when Autopilot and FSD Beta is active?
Q34	Do you typically stay prepared to take corrective actions at all times?
Q35	Has your use of Autopilot (in terms of how you placed your hands on the steering wheel, eyes on the road, and feet) changed over time? If so, how?
